# Magnesium

**Published:** 1831

**Authors:** 


16. *Magnesium*, or the metallic base of magnesia, has been obtained by M. Bussy, of the Academy of Sciences in Paris, by the decomposition of the chloride of magnesium, by means of potassum. The chloride of magnesium may be formed by passing chlorine gas over red hot magnesia; oxygen is expelled from the magnesia, and the chlorine then takes the place of the oxygen, and forms chloride of magnesium. Magnesium is a brilliant metal, of a silvery whiteness, perfectly ductile and malleable, fusible at a comparatively low heat, and like zinc, capable of sublimation at a temperature very little above its fusing point, and of being condensed in form of globules. It does not decompose water at the ordinary temperature; it oxydises at a high temperature, and magnesia is the result; the oxydatum takes place slowly if the pieces be large, but if the metal be in fine powder, it burns with great splendor, throwing out sparks like iron in oxygen gas.—*Journ. R. Ins. May*, 1831.

